# Association Study between Idiopathic Scoliosis and Polymorphic Variants of *VDR*, *IGF-1*, and *AMPD1* Genes

**DOI:** 10.1155/2015/852196

**Published:** 2015-08-25

**Authors:** Svetla Nikolova, Vasil Yablanski, Evgeni Vlaev, Luben Stokov, Alexey Slavkov Savov, Ivo Marinov Kremensky

**Affiliations:** ^1^National Genetic Laboratory, Department of Obstetrics and Gynecology, Faculty of Medicine, Medical University-Sofia, 2 Zdrave Street, 14th Floor, 1431 Sofia, Bulgaria; ^2^Tokuda Hospital Sofia, Orthopedic and Traumatology Clinic, 51B Nikola Vaptsarov Boulevard, 1407 Sofia, Bulgaria; ^3^University Orthopedic Hospital “Prof. Boycho Boychev”, Medical University-Sofia, 56 Nikola Petkov Boulevard, 1614 Sofia, Bulgaria; ^4^Molecular Medicine Center, Medical University-Sofia, 2 Zdrave Street, 14th Floor, 1431 Sofia, Bulgaria

## Abstract

Idiopathic scoliosis (IS) is a complex genetic disorder of the musculoskeletal system, characterized by three-dimensional rotation of the spine with unknown etiology. For the aims of the current study we selected 3 single nucleotide polymorphisms with a low incidence of the polymorphic allele in Bulgarian population, *AMPD1* (rs17602729), *VDR* (rs2228670), and *IGF-1* (rs5742612), trying to investigate the association between these genetic polymorphisms and susceptibility to and progression of IS. The polymorphic regions of the genes were amplified by polymerase chain reaction (PCR). The PCR products were cleaved with the appropriate restriction enzymes. The statistical analysis was performed by Pearson's chi-squared test. A value of *p* < 0.05 was considered to be statistically significant. In conclusion, this case-control study revealed no statistically significant association between the *VDR*, *IGF-1*, and *AMPD1* polymorphisms and the susceptibility to IS or curve severity in Bulgarian patients. Replication case-control studies will be needed to examine the association between these candidate-genes and IS in different populations. The identification of molecular markers for IS could be useful for early detection and prognosis of the risk for a rapid progression of the curve. That would permit early stage treatment of the patient with the least invasive procedures.

## 1. Introduction

The failure of genome-wide association studies to account for most heritability of complex diseases has invoked some considerable discussion on the reasons for “missing heritability” [1]. Factors thought to contribute to this missing heritability are genetic interactions [2] and rare variants [3]. Rare disease variants are thought to exist as recently derived highly penetrant alleles that have a low population frequency but account for high disease susceptibility [4]. The contribution of rare variants to complex diseases has been the topic of much consideration and early investigations have suggested promising results [5].

Idiopathic scoliosis (IS) is a complex genetic disorder of the musculoskeletal system, characterized by three-dimensional rotation of the spine with unknown etiology.

It is known that vitamin D receptor (VDR) plays an important role in the regulation of bone mass development. Adolescence is a critical time for the acquisition of bone mass; around 40% of skeletal mass is acquired during pubertal maturation [6]. Morrison et al. reported that a* VDR Bsm*I polymorphism is responsible for peak bone mass formation [7]. Suh et al. conducted a study between adolescent idiopathic scoliosis (AIS) and the* VDR Fok*I (rs2228670),* Bsm*I (rs1544410), and Cdx2 (rs11568820) gene polymorphisms and found association between the* VDR Bsm*I polymorphism and bone mineral density at the lumbar spine in girls with AIS [8]. Yilmaz et al. found no correlation between the* VDR Bsm*I polymorphism and curve progression [9].

FokI restriction polymorphism could be considered as an independent marker in the* VDR* gene since there is no linkage disequilibrium (LD) with any of the other* VDR* polymorphisms [10]. Therefore, LD with another polymorphism is not a likely explanation for the associations observed with this polymorphism, and so functional studies should be focussed on the polymorphism itself. Two protein variants can exist corresponding to the two polymorphic alleles: a long version of the VDR protein (the T-allele or the “f” allele) and a protein shortened by three amino acids (the C-allele or the “F” allele) [11]. In a study by Arai et al., evidence for the functionality of the FokI polymorphism was obtained. Results from transcriptional activation studies in transfected HeLa cells suggested the short 424-amino-acid VDR protein variant (corresponding with the C-allele or “big F” allele) to be more active than the long 427-amino-acid variant, with a 1.7-fold difference between the two variants [12]. Jurutka et al. demonstrated the 424-amino-acid VDR variant to interact more efficiently with the transcription factor TFIIB. The authors concluded the 424-amino-acid short VDR variant to represent a more transcriptionally potent VDR protein [13].

The importance of insulin-like growth factor-1 (IGF-1) in skeletal growth makes it a good candidate-gene which would play a role in the documented association of rapid growth with curve progression in IS. Yeung et al. found that the* IGF-1* promoter polymorphism rs5742612 affects the curve severity of AIS and may be a disease modifying gene [14]. The irregular expression of growth hormone and insulin-like growth factor-1 (IGF-1) may disturb hormone metabolism, result in a gross asymmetry, and promote the progress of adolescent idiopathic scoliosis [15]. Takahashi et al. concluded that* IGF-1* gene polymorphism rs5742612 is not associated with either AIS predisposition or curve severity in the Japanese [16]. Moon et al. revealed that the* IGF-1* rs5742612 polymorphism is associated with both susceptibility to and curve severity in AIS in Korean patients [17].

The adenosine monophosphate deaminase 1 (AMPD) is expressed in the skeletal muscles, where it plays a role in the production of energy. The nonsense mutation c.34C > T (C to T transition in nucleotide 34, p.Gln12X, rs17602729) in exon 2 of the* AMPD1* gene converts glutamine codon (CAA) into the premature stop codon (TAA), which results in the early interruption of protein synthesis and appears to be the main cause of AMPD deficiency [18]. Studies have shown that part of the population who express the mutant* AMPD1* T-allele (2% of the Caucasian population are homozygous) are vulnerable to muscle weakness, muscular cramps, pain, and premature fatigue during exercises [19].

The effects of scoliosis include poor posture, shoulder humping, muscle weakness, and pain. The* AMPD1* (rs17602729) polymorphism is not investigated in patients with IS.

For the aims of the current study we selected 3 single nucleotide polymorphisms with a low incidence of polymorphic allele in Bulgarian population,* AMPD1* (rs17602729, C/T; C34T),* VDR* (rs2228670, T/C;* Fok*I), and* IGF-1* (rs5742612 C/T), trying to investigate the association between these genetic polymorphisms and susceptibility to and progression of IS.

## 2. Materials and Methods

In this study, Bulgarian patients with IS (*n* = 105) and healthy unrelated gender-matched controls (*n* = 210) were included. All participants in the study were informed about its purpose and were included only after the subjects/families signed their informed consent. Peripheral blood samples were obtained from patients and control subjects. The study protocol was approved by the Ethics Committee of the Medical University-Sofia (number 2987/2012).

### 2.1. Patients

Patients with IS were recruited with the help of orthopaedic surgeons from Tokuda Hospital Sofia and University Orthopedic Hospital “Prof. Boycho Boychev.” The IS diagnosis was confirmed clinically and radiologically. Scoliosis as a phenotypic characteristic like Marfan's syndrome was excluded. The curves were measured by the Cobb method. The mean value of Cobb angles was 54.5 ± 22.6. The mean age at the beginning of the disease was 11.2 ± 3.1 years. In this study, male (*n* = 19) and female (*n* = 86) patients were included.

### 2.2. Controls

The control group including healthy subjects without clinical signs of IS was recruited from a pool of unrelated gender-matched volunteers from other units and clinics of Tokuda Hospital Sofia, National Genetic Laboratory, hospital staff members, and students. The controls were selected among adult patients with skeletal maturity with negative family history of IS. Radiological examination was not performed in the control group.

### 2.3. Genotyping

Genomic DNA was extracted from the peripheral blood leucocytes using magnetic bead technology (chemagic DNA Blood Kit special, Chemagen) on automated high throughput nucleic acid isolation platform (chemagic Magnetic Separation Module I, Chemagen).

The polymorphic regions of the genes were amplified by polymerase chain reaction (PCR). The primer sets are listed in Table 1.

The PCR was carried out in a reaction mix of 20 *µ*L containing 100-ng DNA and 10x Prime Taq buffer (Genet Bio), 10 mM dNTPs Mixture (Genet Bio), 20 pmol Forward and Reverse primers (Alpha DNA), and 0.1 U Prime Taq DNA Polymerase (Genet Bio). PCR amplification was performed in an AB 2720 Thermocycler (Life Technologies) with an initial denaturation at 94°C for five minutes and a final extension of seven minutes at 72°C. The following thermal cycle was repeated 30 times: denaturation at 94°C for 30 seconds, annealing for 30 seconds at temperature presented in Table 2, and extension at 72°C for 30 seconds.

The PCR products were cleaved with the appropriate restriction enzymes (New England Biolabs), according to the manufacturer's instructions, and the restriction fragments were separated on agarose 3% gel in VG-SYS Horizontal Electrophoresis System (Biochrom). The restriction enzymes and the lengths of the fragments representing the genotypes are presented in Table 2.

### 2.4. Statistical Analysis

The statistical analysis was performed by Pearson's chi-squared test to make genotype and allele comparisons between cases and controls and test for Hardy-Weinberg equilibrium. A value of *p* < 0.05 was considered to be statistically significant for comparison between data sets. Odds ratios (OR) were calculated with 95% confidence interval (95% CI). Statistical analysis was conducted with the SPSS 19.0 software package for Windows.

## 3. Results and Discussion

Predisposition for IS, like other examples of complex traits, does not have a specific assigned risk of heritability, but inheritance is based on multiple factors, potentially both genetic and environmental [20]. This study examined the association between IS and* AMPD1* (rs17602729, C/T; C34T),* VDR* (rs2228670, T/C;* Fok*I, F/f), and* IGF-1* (rs5742612 C/T). Genotypes were in Hardy-Weinberg equilibrium.

The overall frequencies of the FF and the ff genotype of the* VDR* (rs2228670) in the patients with IS were comparable with the controls (FF versus Ff + ff, *p* = 0.75). In conclusion, a specific genotype/allele was not associated with a higher risk of scoliosis (FF versus Ff + ff; OR: 1.08; CI: 0.68–1.73; F versus f, *p* = 0.89; OR: 1.02; CI: 0.7–1.49). The genotype and allele frequencies of* IGF-1* (rs5742612) were also comparable between cases and controls (TT versus CC + CT, *p* = 0.71; OR: 1.15; 95% CI: 0.54–2.45; T versus C, *p* = 0.57; OR: 1.22; 95% CI: 0.61–2.45). The genotype and allele frequencies of* AMPD1* (rs17602729) were also comparable between cases and controls (CC versus CT + TT, *p* = 0.6; OR: 0.87; 95% CI: 0.52–1.45; C versus T, *p* = 0.4; OR: 0.83; 95% CI: 0.53–1.29).

On the basis of these results the genetic variants of* VDR*,* IGF-1*, and* AMPD1* alone could not be considered as predisposing factors for IS in Bulgarian population. Previously, Suh et al. found an association between the* VDR Bsm*I polymorphism and bone mineral density in AIS patients but they found no correlation between* Fok*I polymorphism and AIS [8]. Yilmaz et al. reported no statistically significant association between the* VDR* BsmI polymorphism and AIS in Turkish patients [9]. The genetic association between* IGF-1* promoter polymorphism rs5742612 and AIS predisposition was found among Korean [17] but not in Japanese population group [16]. The different results for* IGF-1* could be explained with different genotype and allele frequencies in different population and ethnic groups. Additional case-control studies will be necessary to examine the potential association between other VDR polymorphisms and IS in different population groups. There is no data for previous association studies between C34T* AMPD1* gene polymorphism and IS. Replication studies will be necessary to confirm our results.

The genotype and allele frequencies of the case and the control group are summarised in Table 3.

In the subgroup of surgical cases (*n* = 84) where Cobb angle >40° the genotype and allele frequencies of the* VDR* (rs2228670) polymorphism were comparable between cases and controls (FF versus Ff + ff, *p* = 0.82; OR: 0.94; 95% CI: 0.57–1.57; F versus f, *p* = 0.86; OR: 0.96; 95% CI: 0.65–1.44). The genotype and allele frequencies of the* AMPD1* (rs17602729) polymorphism were also comparable between cases and controls (CC versus TT + CT, *p* = 0.33; OR: 0.76; 95% CI: 0.44–1.32; C versus T, *p* = 0.17; OR: 0.72; 95% CI: 0.45–1.15). In this subgroup,* IGF-1* (rs5742612) CC genotype was not observed (TT versus CC + CT, *p* = 1; OR: 1; 95% CI: 0.46–2.18; T versus C, *p* = 0.67; OR: 1.17; 95% CI: 0.56–2.46).

In conclusion, these polymorphisms could not be considered as modifying factors of IS associated with a rapid progression of the deformity in Bulgarian population. First, Yeung et al. found that the* IGF-1* promoter polymorphism rs5742612 affects the curve severity of AIS and may be a disease modifying gene in Chinese population [14]. Takahashi et al. concluded that* IGF-1* gene polymorphism rs5742612 is not associated with either AIS predisposition or curve severity in Japanese population [16]. Moon et al. revealed that the* IGF-1* rs5742612 polymorphism is associated with both susceptibility to and curve severity in AIS in Korean patients [17]. On the basis of these results* IGF-1* remains a candidate-gene of IS and replication studies are necessary to investigate the contribution of the* IGF-1* polymorphic variants in the etiopathogenesis of IS in different population groups.

Adolescent idiopathic scoliosis (AIS) is the most common spinal deformity [21] and the most frequently studied idiopathic scoliosis [8, 9, 14, 16, 17]. In the subgroup of the adolescents (*n* = 78) the genotype and allele frequencies of the* VDR* (rs2228670) polymorphism were comparable between cases and controls (FF versus Ff + ff, *p* = 0.72; OR: 1.1; CI: 0.65–1.86; F versus f, *p* = 0.92; OR: 1.02; 95% CI: 0.67–1.55). The genotype and allele frequencies of the* IGF-1* (rs5742612) polymorphism were comparable between cases and controls (TT versus CC + CT, *p* = 0.84; OR: 0.92; 95% CI: 0.42–2.01; C versus T, *p* = 1; OR: 0.98; 95% CI: 0.48–2.01). The genotype and allele frequencies of the* AMPD1* (rs17602729) polymorphism were also comparable between cases and controls (CC versus TT + CT, *p* = 0.6; OR: 0.86; CI: 0.49–1.51; C versus T, *p* = 0.5; OR: 0.84; 95% CI: 0.51–1.38). These results showed the polymorphisms are not associated with the susceptibility to AIS and confirmed the negative association between the FokI polymorphism and AIS [8] and* IGF-1* (rs5742612) and AIS [16].

In the subgroup of the familial cases (*n* = 28) the genotype and allele frequencies of the* VDR* (rs2228670) were comparable between cases and controls (FF versus Ff + ff, *p* = 0.36; OR: 1.46; 95% CI: 0.65–3.27; F versus f, *p* = 0.58; OR: 1.2; CI: 0.62–2.32). The genotype and allele frequencies of the* IGF-1* (rs5742612) were comparable between cases and controls (TT versus CC + CT, *p* = 0.55; OR: 1.76; 95% CI: 0.39–7.85; T versus C, *p* = 0.79; OR: 1.31; 95% CI: 0.39–4.45). The genotype and allele frequencies of* AMPD1* (rs17602729) were also comparable between cases and controls (CC versus TT + CT, *p* = 0.13; OR: 2.29; 95% CI: 0.76–6.88; C versus T, *p* = 0.11; OR: 2.29; CI: 0.8–6.57). In conclusion, the genotypes and alleles of these polymorphisms could not be associated with the familial history of IS.

Scoliosis is more common in females than males. In the subgroup of female patients (*n* = 86) no statistically significant associations between the* VDR*,* IGF-1*, and* AMPD1* polymorphisms and the clinical phenotype were observed (*AMPD1* (rs17602729), CC versus TT + CT, *p* = 0.92, and C versus T, *p* = 0.86;* VDR* (rs2228670), FF versus Ff + ff, *p* = 0.48, and F versus f, *p* = 0.58;* IGF-1* (rs5742612), TT versus CC + CT, *p* = 0.59, and T versus C, *p* = 0.38). In the subgroup of male patients (*n* = 19) no statistically significant associations were observed (*AMPD1* (rs17602729), CC versus TT + CT, *p* = 0.32, and C versus T, *p* = 0.12;* VDR* (rs2228670), FF versus Ff + ff, *p* = 0.45, and F versus f, *p* = 0.35;* IGF-1* (rs5742612), TT versus CC + CT, *p* = 1, and T versus C, *p* = 0.68). In conclusion, the genotypes and alleles of* VDR*,* IGF-1*, and* AMPD1* polymorphisms could not be associated with gender.

Main thoracic curve was the most common curve type in male and female patients as well as surgical cases. In the subgroup of patients with thoracic (*n* = 62), lumbar (*n* = 12), and thoracolumbar (*n* = 31) scoliosis no association between the polymorphisms and the clinical phenotype was observed (*p* > 0.05). In conclusion, these genetic variants were not associated with the curve pattern.

Odds ratios of genotypes and alleles in the subgroups are summarised in Table 4.

The results of the statistical analysis in this study indicate that the* VDR*,* IGF-1*, and* AMPD1* polymorphisms were not associated with susceptibility to IS, curve severity, curve pattern, onset of the disease, familial history, or gender. On the basis of these results the examined polymorphic variants could not be considered as genetic variants with predisposing or modifying effect in Bulgarian population. These results do not exclude a potential role of the same polymorphic markers in other population groups or impact of other polymorphisms of* VDR*,* IGF-1*, and* AMPD1* on the etiology and pathogenesis of IS in Caucasian population.

## 4. Conclusions

In conclusion, this case-control study revealed no statistically significant association between the* VDR*,* IGF-1*, and* AMPD1* polymorphisms and the susceptibility to IS or curve severity in Bulgarian patients.

No genotype or allele of the* VDR*,* IGF-1*, and* AMPD1* polymorphisms was found to be correlated with familial history, age, gender, or curve pattern.

Replication case-control studies will be needed to examine the association between these candidate-genes and IS in different populations.

The identification of molecular markers for IS could be useful for early detection and prognosis of the risk for a rapid progression of the curve. That would permit early stage treatment of the patient with the least invasive procedures.

## Figures and Tables

**Table 1:** PCR primers.

Gene, polymorphism	Primers
*VDR* (rs2228670)	Forward: 5′-AGCTGGCCCTGGCACTGACTCTGCTCT-3′
	Reverse: 5′-ATGGAAACACCTTGCTTCTTCTCCCTC-3′
*IGF-1* (rs5742612)	Forward: 5′-CACACACACAGGTTTGAGTTATATG-3′
	Reverse: 5′-GGTAAAGTAGATTGGAAGACAGC-3′
*AMPD1* (rs17602729)	Forward: 5′-CTCTGACAAATGGCAGCAAA-3′
	Reverse: 5′-TGTCTACCCCAAAGCAGTGA-3′

**Table 2:** PCR-RFLP protocol.

Gene, polymorphism	Annealing temperature, °C	PCR product size, bp	Restriction enzyme	Restriction fragments, bp
*VDR *(rs2228670)	59	265	*Fok*I	FF: 265 Ff: 265 + 196 + 69 ff: 196 + 69

*IGF-1* (rs5742612)	59	330	*Bsl*I	TT: 330 CT: 330 + 277 + 53 CC: 277 + 53

*AMPD1* (rs17602729)	58	136	*Mae*II	CC: 97 + 39 CT: 136 + 97 + 39 TT: 136

PCR indicates polymerase chain reaction; RFLP: restriction fragment length polymorphism; bp: base pair; *VDR*: vitamin D receptor; *IGF-1*: insulin-like growth factor-1; *AMPD1*: adenosine monophosphate deaminase 1.

**Table 3:** Genotype and allele frequency distributions in patients (*n* = 105) with idiopathic scoliosis (IS) and healthy controls (*n* = 210).

Gene, polymorphism	Genotype, allele	Cases *n*, %	Controls *n*, %
*VDR* (rs2228670)	FF	56 (53.3)	108 (51.4)
	Ff	43 (41.0)	92 (43.8)
	ff	6 (5.7)	10 (4.8)
	F	155 (73.8)	308 (73.3)
	f	55 (26.2)	112 (26.7)

*IGF-1* (rs5742612)	TT	94 (89.5)	185 (88.1)
	CT	10 (9.5)	21 (10.0)
	CC	1 (1.0)	4 (1.9)
	T	198 (94.3)	391 (93.1)
	C	12 (5.7)	29 (6.9)

*AMPD1* (rs17602729)	CC	73 (69.5)	152 (72.4)
	CT	27 (25.7)	53 (25.2)
	TT	5 (4.8)	5 (2.4)
	C	173 (82.4)	357 (85.0)
	T	37 (17.6)	63 (15.0)

All *p* values were not significant. *VDR* indicates vitamin D receptor; *IGF-1*: insulin-like growth factor-1; *AMPD1*: adenosine monophosphate deaminase 1.

**Table 4:** Odds ratios of genotypes and alleles in different subgroups with IS.

Subgroup	Gene	Genotype, allele	OR [95% CI]
General (*n* = 105)	*VDR*	FF versus Ff + ff F versus f	1.08 [0.68–1.73] 1.02 [0.70–1.49]
	*IGF-1*	TT versus CC + CT T versus C	1.15 [0.54–2.45] 1.22 [0.61–2.45]
	*AMPD1*	CC versus TT + CT C versus T	0.87 [0.52–1.45] 0.83 [0.53–1.29]

AIS (*n* = 78)	*VDR*	FF versus Ff + ff F versus f	1.10 [0.65–1.86] 1.02 [0.67–1.55]
	*IGF-1*	TT versus CC + CT T versus C	0.92 [0.42–2.01] 0.98 [0.48–2.01]
	*AMPD1*	CC versus TT + CT C versus T	0.86 [0.49–1.51] 0.84 [0.51–1.38]

Familial history of IS (*n* = 28)	*VDR*	FF versus Ff + ff F versus f	1.46 [0.65–3.27] 1.20 [0.62–2.32]
	*IGF-1*	TT versus CC + CT T versus C	1.76 [0.39–7.85] 1.31 [0.39–4.45]
	*AMPD1*	CC versus TT + CT C versus T	2.29 [0.76–6.88] 2.29 [0.80–6.57]

Cobb angle >40° (*n* = 84)	*VDR*	FF versus Ff + ff F versus f	0.94 [0.57–1.57] 0.96 [0.65–1.44]
	*IGF-1*	TT versus CC + CT T versus C	1.00 [0.46–2.18] 1.17 [0.56–2.46]
	*AMPD1*	CC versus TT + CT C versus T	0.76 [0.44–1.32] 0.72 [0.45–1.15]

Thoracic scoliosis (*n* = 62)	*VDR*	FF versus Ff + ff F versus f	1.50 [0.84–2.67] 1.25 [0.78–2.00]
	*IGF-1*	TT versus CC + CT T versus C	1.06 [0.44–2.59] 1.24 [0.53–2.90]
	*AMPD1*	CC versus TT + CT C versus T	1.07 [0.57–2.01] 1.03 [0.59–1.79]

Lumbar scoliosis (*n* = 12)	*VDR*	FF versus Ff + ff F versus f	1.32 [0.41–4.30] 1.09 [0.42–2.82]
	*IGF-1*	TT versus CC + CT T versus C	1.48 [0.31–7.15] 1.93 [0.54–6.84]
	*AMPD1*	CC versus TT + CT C versus T	1.91 [0.41–8.97] 1.24 [0.36–4.26]

Thoracolumbar scoliosis (*n* = 31)	*VDR*	FF versus Ff + ff F versus f	1.93 [0.88–4.22] 1.41 [0.80–2.49]
	*IGF-1*	TT versus CC + CT T versus C	1.96 [0.44–8.72] 2.23 [0.52–9.57]
	*AMPD1*	CC versus TT + CT C versus T	1.66 [0.76–3.62] 1.81 [0.95–3.43]

Males (*n* = 19)	*VDR*	FF versus Ff + ff F versus f	0.65 [0.22–1.98] 0.65 [0.27–1.60]
	*IGF-1*	TT versus CC + CT T versus C	0.73 [0.11–4.78] 0.65 [0.14–3.05]
	*AMPD1*	CC versus TT + CT C versus T	0.56 [0.18–1.77] 0.47 [0.18–1.24]

Females (*n* = 86)	*VDR*	FF versus Ff + ff F versus f	1.21 [0.72–2.03] 1.13 [0.74–1.71]
	*IGF-1*	TT versus CC + CT T versus C	1.25 [0.55–2.86] 1.42 [0.65–3.11]
	*AMPD1*	CC versus TT + CT C versus T	0.97 [0.54–1.73] 0.96 [0.58–1.58]

All *p* values were not significant. IS indicates idiopathic scoliosis; AIS: adolescent idiopathic scoliosis; OR: odds ratio; CI: confidence interval; *VDR*: vitamin D receptor; *IGF-1*: insulin-like growth factor-1; *AMPD1*: adenosine monophosphate deaminase 1.
